# How does pharmacological and toxicological knowledge evolve? A case study on hydrogen cyanide in German pharmacology and toxicology textbooks from 1878 to 2020

**DOI:** 10.1007/s00210-024-03227-z

**Published:** 2024-06-20

**Authors:** Laureen Ludwig, Roland Seifert

**Affiliations:** Institute of Pharmacology, Hannover Medical School, Carl-Neuberg-Straße 1, 30625 Hannover, Germany

**Keywords:** Hydrogen cyanide, Poison, Textbook analysis, Content analysis, History of pharmacology and toxicology

## Abstract

Little is known about how pharmacological and toxicological knowledge evolves. The aim of this study was to investigate the changes in the presentation of the poison hydrogen cyanide in sixteen German-language pharmacology and toxicology textbooks from 1878 to 2020. The categories of structure, molecular mechanism of action, occurrence, effects, resorption, areas of application, lethal dose, acute symptoms of intoxication, treatment of hydrogen cyanide poisoning, and recommended therapeutic preparations were evaluated. The knowledge on the structure, lethal dosage, and occurrence of hydrogen cyanide has remained constant. In contrast, knowledge on molecular mechanism of action and recommended preparations of the poison has changed dramatically. Until 1944, the binding of hydrogen cyanide to hemoglobin was considered the mechanism of action, whereas from 1951 onwards, the interaction of hydrogen cyanide with the Fe^3+^ of cytochrome oxidase was described. The number of preparations containing hydrogen cyanide decreased into obsolescence until 1951. The areas of application of hydrogen cyanide also show a change, as from 1919 onwards, mainly industrial areas of application of the poison are described instead of medical ones, and from 1951 onwards, criminalistic areas of application are also mentioned. Thus, pharmacological and toxicological knowledge develops non-linearly, molecular mechanism and uses being the most dynamic areas, whereas the knowledge about hydrogen cyanide’s chemical structure, lethal dose, and occurrence remained constant. Older pharmacology and toxicology textbooks were better than newer ones at discussing changes in scientific concepts. Pharmacology and toxicology textbooks also mostly failed to discuss the misuse of hydrogen cyanide (Zyklon B) during the Nazi regime, missing an important opportunity to showcase the ethical responsibility of pharmacology and toxicology. Thus, future pharmacology and toxicology textbooks should improve on discussing the development of pharmacological and toxicological concepts and the ethical responsibility of the discipline.

**Supplementary Information:**

The online version contains supplementary material available at 10.1007/s00210-024-03227-z.

## Introduction

Medical students use pharmacology and toxicology textbooks to prepare for examinations in medical school, which is why the contents presented in the books on therapeutic and adverse effects, indications, and contraindications of drugs form the knowledge base of future doctors. However, little is known about how pharmacological knowledge develops and changes over time in textbooks. The importance of portraying current knowledge and clinical practice in textbooks is shown by the correlation between insufficient intravenous fluid prescribing knowledge and practices by junior doctors in the UK and inadequate treatment of the topic in medical textbooks (Powell et al. 2014). A recent analysis of German-language pharmacology and toxicology textbookson the antihypertensive drug reserpine has revealed that they are substantially lagging clinical practice (Misera and Seifert 2023). An evaluation of the portrayal of a specific drug or poison over the entire history of pharmacology has not yet taken place. Therefore, in this case study, we assessed the presentation of hydrogen cyanide based on sixteen German-language textbooks over almost 150 years.

For interpretation of the history, current knowledge on hydrogen cyanide is presented below. Hydrogen cyanide (HCN) is a weak acid that forms water-soluble salts (cyanides) on contact with alkalis, including potassium cyanide (KCN) and sodium cyanide (NaCN) (Aktories et al. 2022). Hydrogen cyanide can be absorbed via the respiratory tract, gastrointestinal tract, or the skin and mucous membranes (Graham and Traylor 2024). Hydrogen cyanide binds to the trivalent iron of cytochrome oxidase, which is part of the mitochondrial respiratory chain, and thus inhibits cellular respiration and the production of ATP (Graham and Traylor 2024). As a result, cellular hypoxia develops and ATP concentration decreases, causing metabolic acidosis (Graham and Traylor 2024). Hydrogen cyanide is metabolized by rhodanese, which is primarily found in the liver and muscle, and the inactive metabolite is subsequently eliminated renally (Graham and Traylor 2024). HCN is naturally found in bitter almonds and in the kernels of stone fruits such as apricots, peaches, and plums, as well as in lima beans (Bolarinwa et al. 2016; Graham and Traylor 2024). Hydrogen cyanide intoxications can occur through the inhalation of combustion gases or as part of therapy with sodium nitroprusside (Brunton and Knollmann 2023; Graham and Traylor 2024). The current use of hydrogen cyanide is limited to non-medical applications such as the chemical and metal industries, for example for galvanization and steel hardening, to produce blue dyes, in photography, and as a pesticide (Marquardt et al. 2019; Graham and Traylor 2024). HCN can also be misused in criminal applications (murder, mass murder and suicide) (Marquardt et al. 2019; Graham and Traylor 2024). During the Second World War (1939–1945), hydrogen cyanide was used by the Nazis under the name Zyklon B for the genocide of Jews in the gas chambers of concentration camps (Embar-Seddon and Pass 2009; Graham and Traylor 2024). Symptoms of acute hydrogen cyanide poisoning include a bitter smell of bitter almonds when inhaled, headache, dizziness, confusion, tachypnea, and tachycardia, dyspnea, and apnea up to coma and death (Hendry-Hofer et al. 2019; Graham and Traylor 2024). Hydroxocobalamine is considered the treatment of first choice for hydrogen cyanide poisoning (Aktories et al. 2022). Dimethylaminophenol can also be used for therapeutic induction of met-hemoglobin in cases of intoxication (Aktories et al. 2022). Sodium thiosulfate can be given supportively to accelerate the body’s own detoxification by providing sulfur (Marquardt et al. 2019; Aktories et al. 2022). Ventilation with oxygen is indicated as part of the symptomatic treatment of hydrogen cyanide intoxication (Marquardt et al. 2019). The administration of sodium hydrogen carbonate is also suitable for correcting the metabolic acidosis (Marquardt et al. 2019).

## Material and methods

### Selection of textbooks

One textbook per decade was analyzed as an example to compare the content presented for hydrogen cyanide in pharmacology and toxicology textbooks from 1878 onwards (Table 1). The selection criteria included that the textbooks must be intended for medical students and doctors and be published in German language. A further selection criterion was the availability of the textbooks.

**Table 1:** Pharmacology and toxicology textbooks used for the analysis. All textbooks analyzed are listed with the name of the editor, edition, reference, total number of pages, and page references from the index relating to hydrogen cyanide and its cyanides. If a page is given in the index, but hydrogen cyanide or its cyanides are not explicitly dealt with on the corresponding page, the page reference is not listed in the table. Pages on which the content of hydrogen cyanide is continued from preceding or neighboring specified pages are also listed as individual pages in the table. This also applies to pages that are labelled as subsequent pages in the index

ID number	Textbook group	Author	Year of publication	Edition	Reference	Pages	Hydrogen cyanide-related pages (keyword index)
1	1	Buchheim	1878	3	Buchheim (1878)	618	197, 198, 199, 200, 201, 202
2	1	Schmiedeberg	1883	1	Schmiedeberg (1883)	279	52, 53
3	1	Husemann	1892	3	Husemann (1892)	705	585, 586, 587, 588, 589
4	1	Filehne	1901	10	Filehne (1901)	421	15, 94, 119, 267
5	2	Tappeiner	1919	13	Tappeiner (1919)	499	259, 260
6	2	Schmiedeberg	1921	8	Schmiedeberg (1921)	657	92, 93, 94, 95, 96, 97
7	2	Hoesslin, Müller	1933	4	Hoesslin and Müller (1933)	245	26, 34, 35, 87, 154, 155, 217
8	2	Eichholtz	1944	3 and 4	Eichholtz (1944)	525	194, 245, 317, 321, 447, 448, 469, 475
9	3	Eichholtz	1951	7	Eichholtz (1951)	594	12, 60, 200, 208, 274, 336, 349, 440, 465, 471, 473, 475, 476, 499
10	3	Kuschinsky, Lüllmann	1964	1	Kuschinsky and Lüllmann (1964)	331	276, 277
11	3	Forth, Henschler, Rummel	1977	2	Forth et al. (1977)	672	582, 583
12	3	Estler	1986	Supp study edition	Estler (1986)	648	68, 542, 543, 544, 587
13	4	Oberdisse, Hackenthal, Kuschinsky	1997	1	Oberdisse et al. (1997)	770	729, 730
14	4	Aktories, Förstermann, Hofmann, Starke	2005	9	Aktories et al. (2005)	1189	761, 1006, 1008, 1009, 1010, 1051, 1059, 1060, 1061, 1062, 1081
15	4	Graefe, Lutz, Bönisch	2016	2	Graefe et al. (2016)	836	714, 766, 767, 769
16	4	Freissmuth, Offermanns, Böhm	2020	3	Freissmuth et al. (2020)	1064	947, 948, 511

### Analyzing the data

**Fig. 1:**
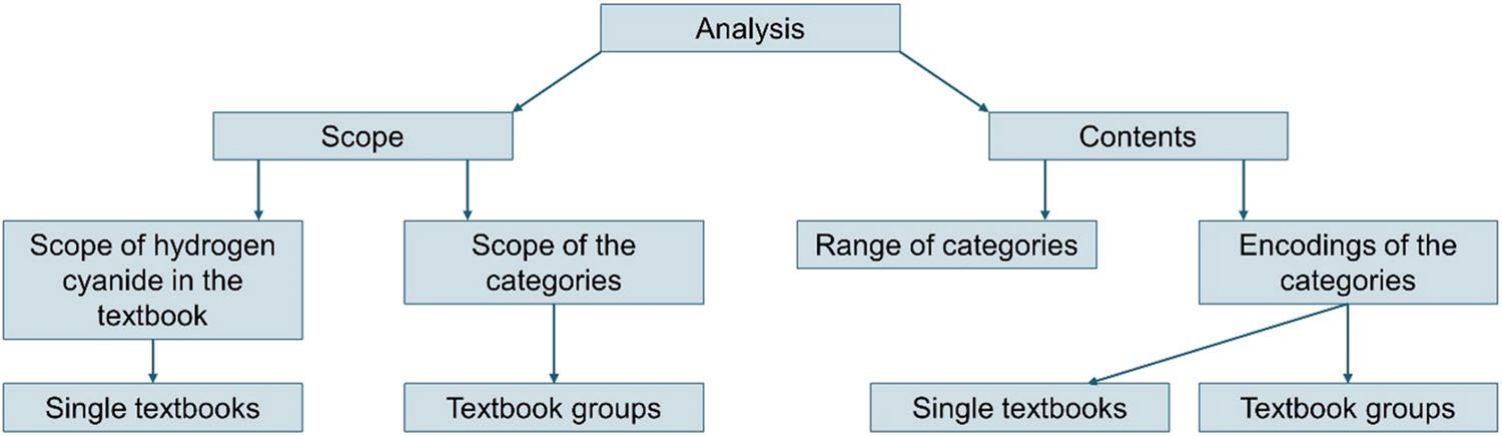
Methodological procedure for analyzing the data. For a detailed list of the encodings, see supplemental figures S1–S10

Figure 1 illustrates the methodological approach used to examine the data. Tables S1–S10 show the analysis categories with encodings and the detailed results for the textbook groups. The scope of hydrogen cyanide-related pages in the pharmacology and toxicology textbooks was analyzed. The scope of the categories (structure, molecular mechanism of action, occurrence, effects, resorption, areas of application, lethal dose, acute symptoms of intoxication, treatment of hydrogen cyanide poisoning, and recommended therapeutic preparations) was determined. The pharmacology and toxicology textbooks were divided into textbook groups chronologically: 1878–1901 (Textbook group 1), 1919–1944 (Textbook group 2), 1951–1986 (Textbook group 3), 1997–2020 (Textbook group 4)*.*

The content of the textbooks was analyzed. The average range of the categories was calculated. An inductive approach was chosen to digitize the data. This procedure makes it possible, after the verbatim transfer of the primary information from the textbooks into the associated categories and subsequent definition of the encodings, to present all the collected content in a comparable form. All encodings within an analysis category were assigned numbers, which in turn were assigned to the textbooks that listed the contents of the corresponding encodings.

## Results and discussion

### Scope of hydrogen cyanide-related content in pharmacology and toxicology textbooks

**Fig. 2:**
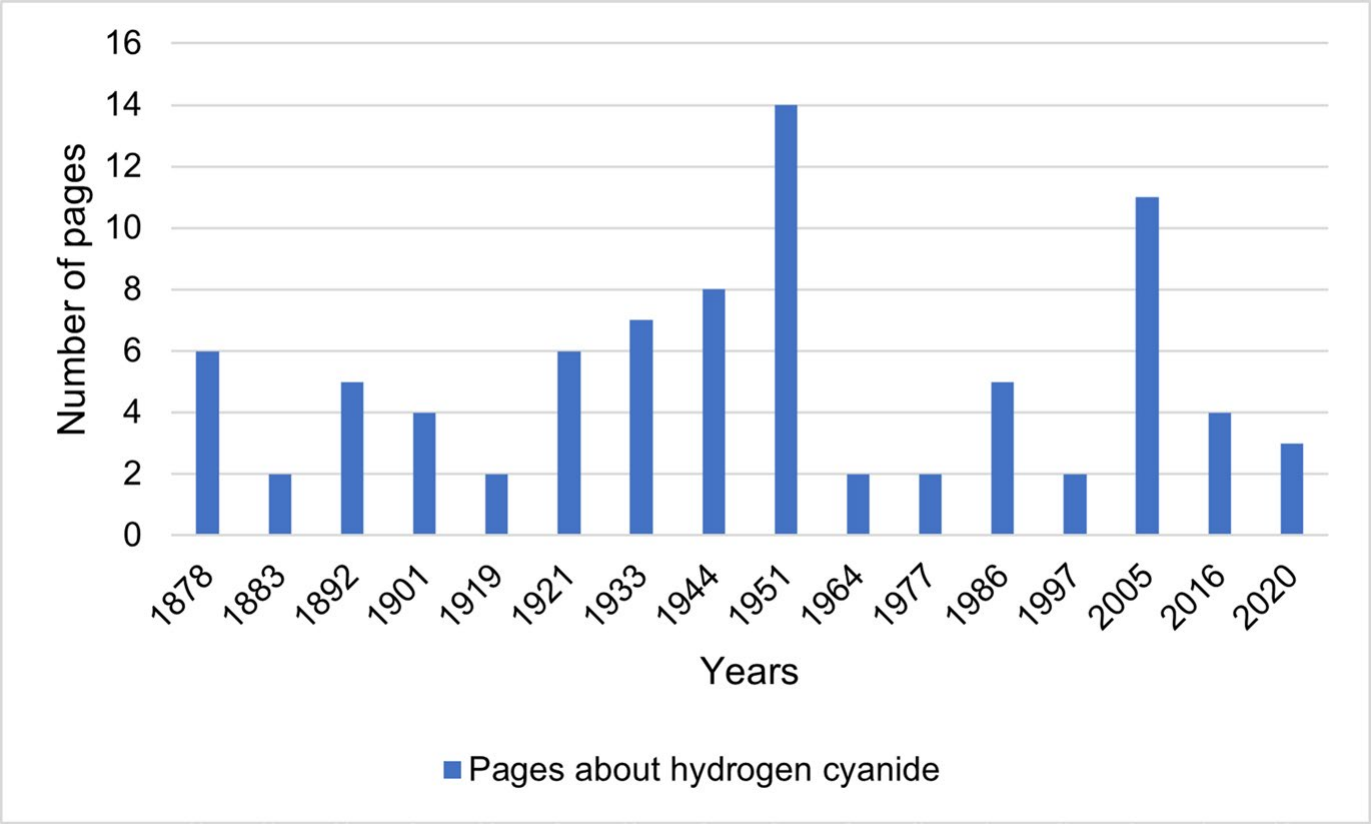
Number of pages with content on hydrogen cyanide and its cyanides. The number of pages with content on hydrogen cyanide is based on the page references from the index, which are related to hydrogen cyanide or its cyanides and are listed in Table 1. If a page is listed in the index, but hydrogen cyanide or its cyanides are not explicitly discussed on the corresponding page, the page reference is not listed in the table and included in the analysis. Pages on which the content on hydrogen cyanide is continued from previous or neighboring specified pages are also listed as individual pages in the table and counted in the analysis. This also applies to pages that are labelled as subsequent pages in the index

**Fig. 3:**
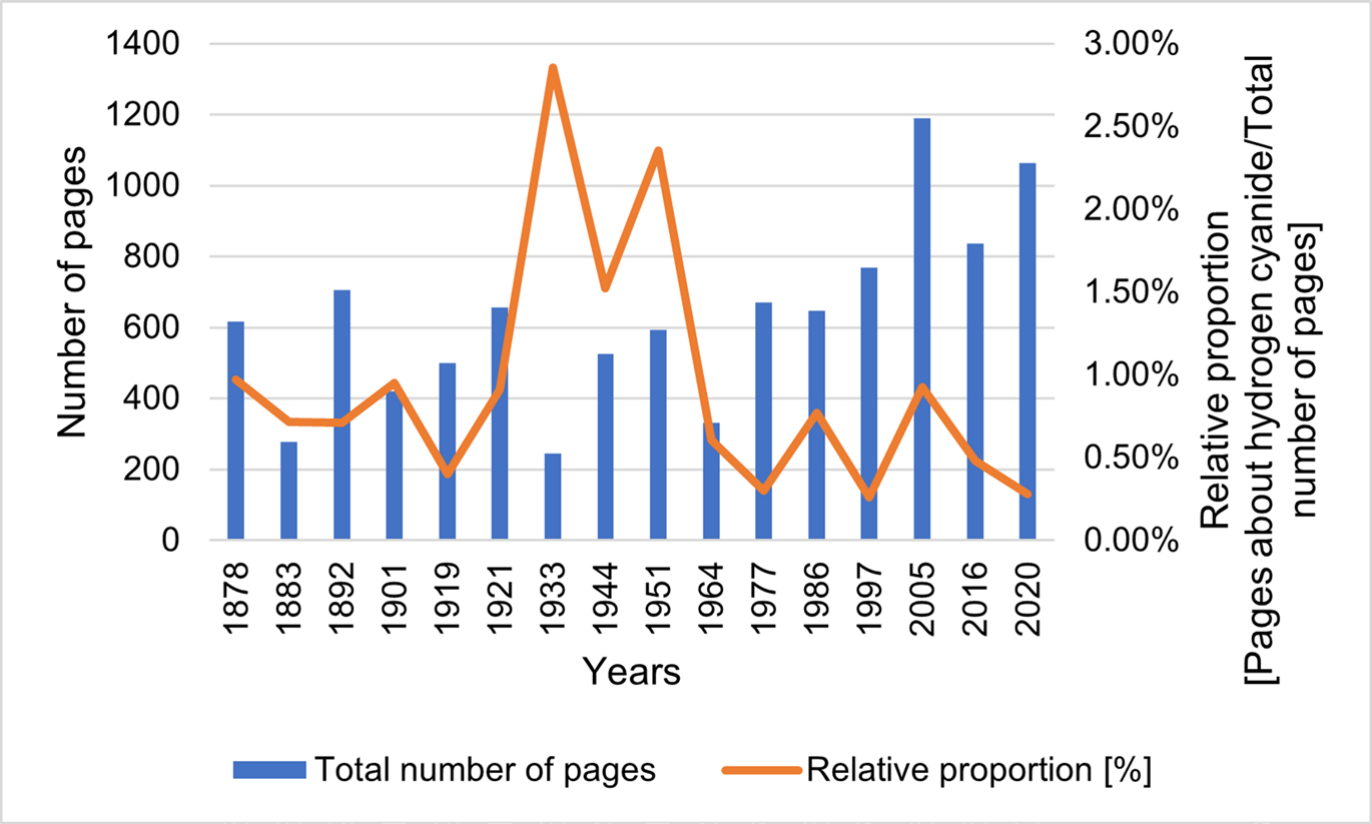
Total number of pages in the textbooks and relative share of poison pages in the total number of pages in the textbook

Figure 2 shows the number of pages with content on hydrogen cyanide and its cyanides. Figure 3 shows the total number of pages in the textbooks and the relative proportion of poison pages in the total number of pages in the textbook. The number of pages with hydrogen cyanide-related content reaches its maximum in textbook 9 in 1951 with 14 pages and then drops to 2 pages in textbook 10 in 1964. In the period 1964–2020, the number of substance pages is at a low-medium level. The relative share of poison pages in the total number of pages in the textbooks reaches its maximum in textbook 7 in 1933 at 2.86% and shows a further peak in textbook 9 in 1951 at 2.36%. A connection between the increased representation of the poison in the period 1933–1951 and the use of hydrogen cyanide in the form of Zyklon B as a lethal poison in Nazi concentration camps during the Second World War (1939–1945) is possible (Embar-Seddon and Pass 2009). To test this hypothesis, the textbook “Grundriß der Pharmakologie, Toxikologie (Wehr-Toxikologie) und Arznei-Verordnungslehre” (“Principles of Pharmacology, Toxicology (Military Toxicology) and Drug Prescription”) by the German pharmacologist and NSDAP member Heinrich Gebhardt from 1940 was examined (Philippu and Seifert 2023). But here, hydrogen cyanide is only sketchily presented (4 of 403 pages, 1%) (Gebhardt, 1940).

### Range of categories on hydrogen cyanide

**Fig. 4:**
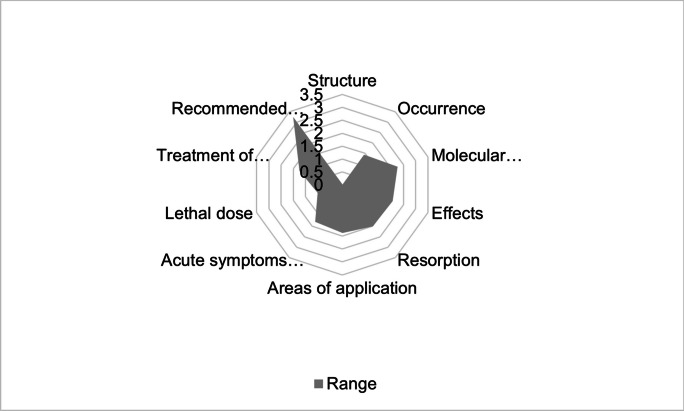
Range of all categories. The calculation of a category’s range is based on the average range of all encodings within this category. The range of individual encodings results from the difference between the highest number of entries per textbook group and the lowest number. The number of possible entries and therefore the range of an encoding varies between 0 and 4. This is based on the number of textbooks per textbook group

Figure 4 shows the range of the categories presented in the textbook groups. The categories *Recommended therapeutic preparations*, *Molecular mechanism of action*, *Effects, Resorption*, *Areas of application*, *Acute symptoms of intoxication*, and *Treatment of hydrogen cyanide poisoning* show an above-average range. Therefore, the change of knowledge is the greatest here. A below-average range was noted in the categories *Occurrence*, *Lethal dose*, and *Structure*. Thus, changes in knowledge on poisoning symptoms, occurrence, lethal dose, and structure mentioned are small.

## Structure of hydrogen cyanide

**Fig. 5:**
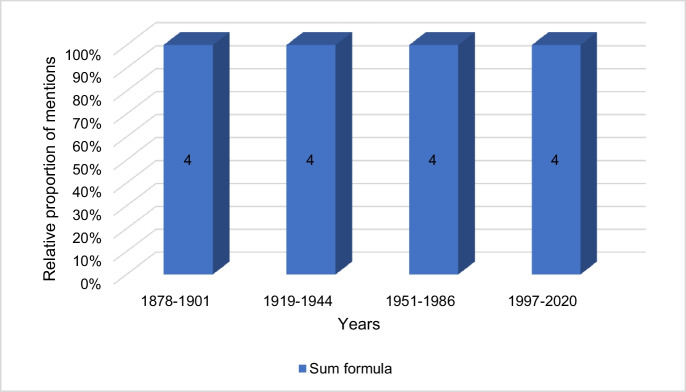
Information on the structure of hydrogen cyanide. The number of entries per encodings is given as a percentage of the total number of entries per textbook group

Figure 5 shows the information on the structure of hydrogen cyanide by textbook group. No change in the information was determined over the course of the study period.

### Molecular mechanism of action of hydrogen cyanide

**Fig. 6:**
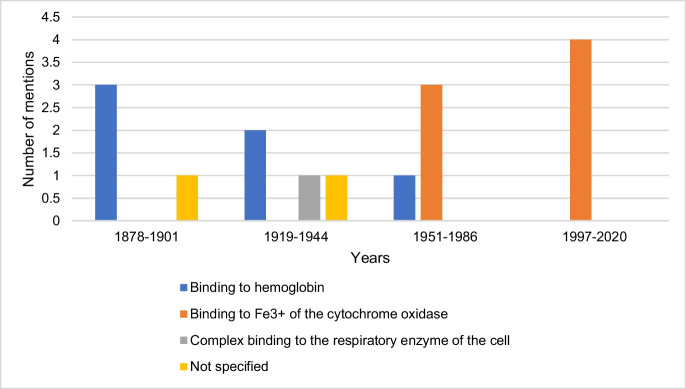
Information on the molecular mechanism of action of hydrogen cyanide. The absolute number of mentions is shown

Figure 6 shows the information on the molecular mechanism of action of hydrogen cyanide by textbook group. Seventy-five percent of the textbooks of the first textbook group and 50% of the second textbook group mention hydrogen cyanide binding to hemoglobin as the molecular mechanism of action of the toxin. Binding to Fe^3+^ of cytochrome oxidase is described in the third textbook group (75%) and fourth textbook group (100%) as the mechanism of action of hydrogen cyanide. This suggests an increase in knowledge regarding the molecular mechanism of action of hydrogen cyanide from the second to the third textbook group.

### Occurrence of hydrogen cyanide

**Fig. 7:**
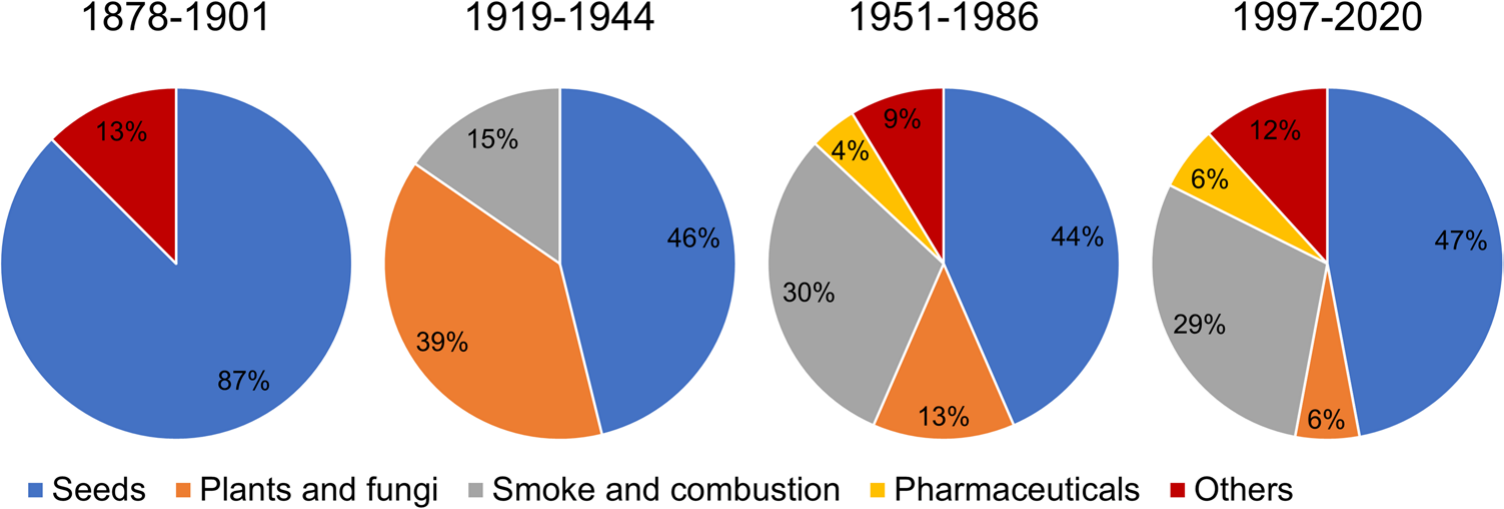
Data on the occurrence of hydrogen cyanide. The absolute and relative number of mentions per textbook group is shown

Figure 7 shows the mentions of the occurrence of hydrogen cyanide by textbook group. The textbooks in the first textbook group cite two different sources of hydrogen cyanide. The information in the second textbook group can be assigned to three different types of occurrences. The textbooks in the third textbook group provide information on five sources of hydrogen cyanide. Among the textbooks analyzed in the fourth textbook group, five different sources of hydrogen cyanide and its cyanides are listed. Overall, a trend towards increasing heterogenization of the content presented in the category *Occurrence* can be identified. However, the occurrence of hydrogen cyanide in seeds is listed most frequently in all textbook groups, which is why the content focus remains unchanged in the period 1878–2020.

### Pharmacological and toxicological effects of hydrogen cyanide

**Fig. 8:**
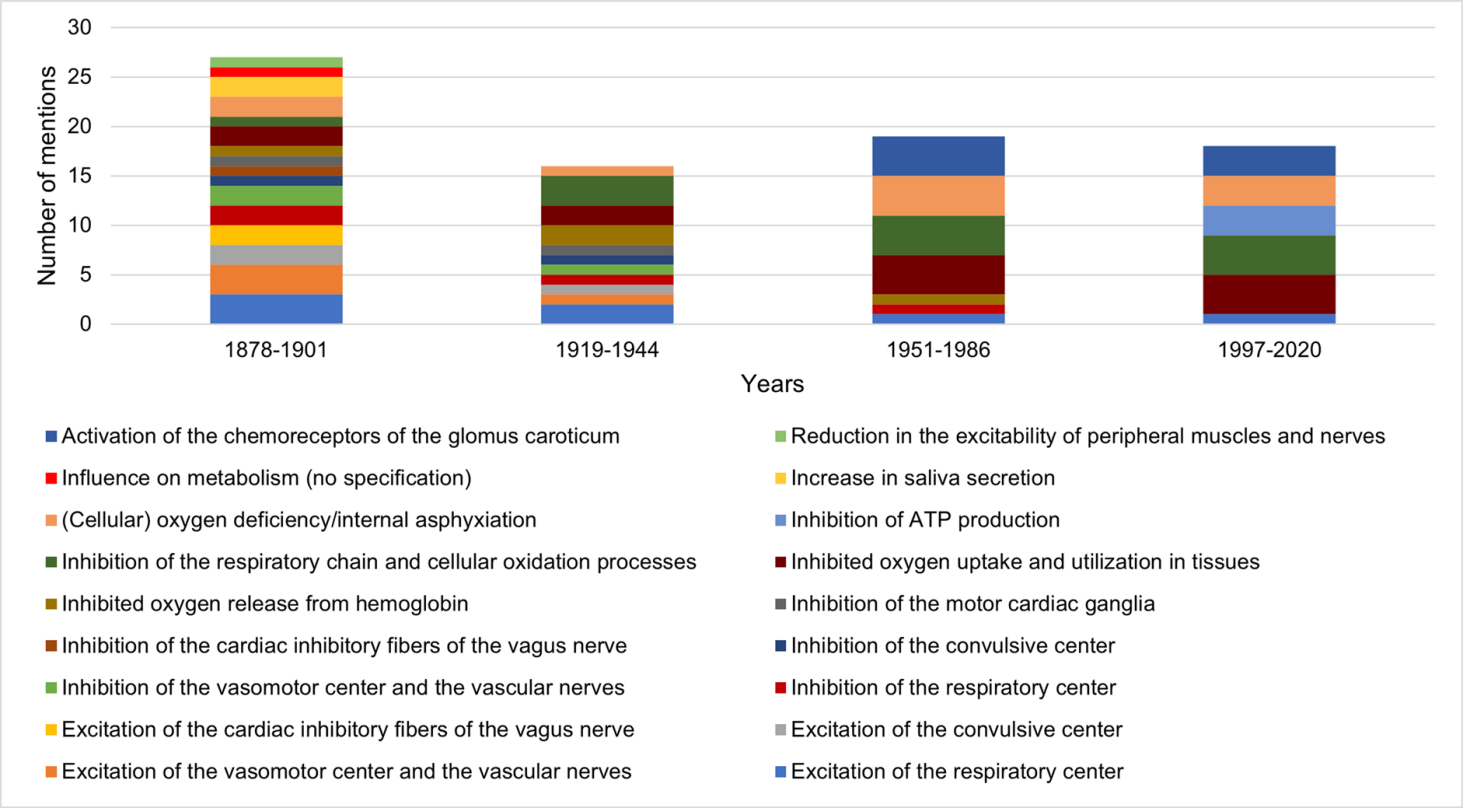
Information on the effects of hydrogen cyanide. The absolute number of mentions is shown

Figure 8 shows the data on the effects of hydrogen cyanide by textbook group. A decrease in the scope of the effects occurred from the first to the second textbook group. From the second textbook group onwards, an increasingly homogenized presentation of the content with a focus on the hydrogen cyanide effects of *Inhibited oxygen uptake and utilization in tissues* and *Inhibition of the respiratory chain and cellular oxidation processes* was noted.

### Resorption of hydrogen cyanide

**Fig. 9:**
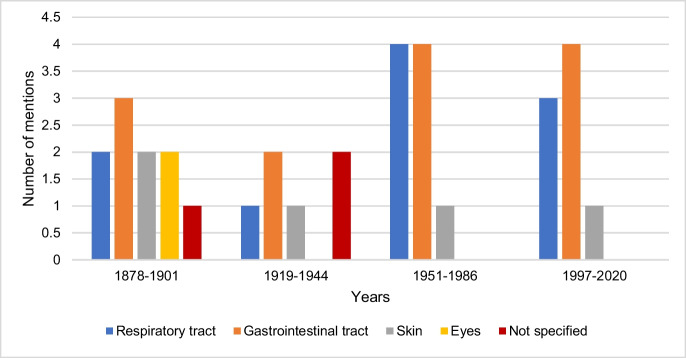
Data on the resorption of hydrogen cyanide. The absolute number of mentions is shown

Figure 9 shows the data on the resorption of hydrogen cyanide by textbook groups. The first textbook group mentions a total of 4 different resorption pathways. From 1919 onwards, there is no further mention of the conjunctival resorption route, which is why the information from the second, third, and fourth textbook groups can each be assigned to 3 different resorption pathways. In the third textbook group, resorption from the gastrointestinal tract and the respiratory tract are described as the most frequent routes of hydrogen cyanide uptake. In the fourth textbook group, the gastrointestinal tract is the most frequently mentioned resorption route.

### Areas of application of hydrogen cyanide

**Fig. 10:**
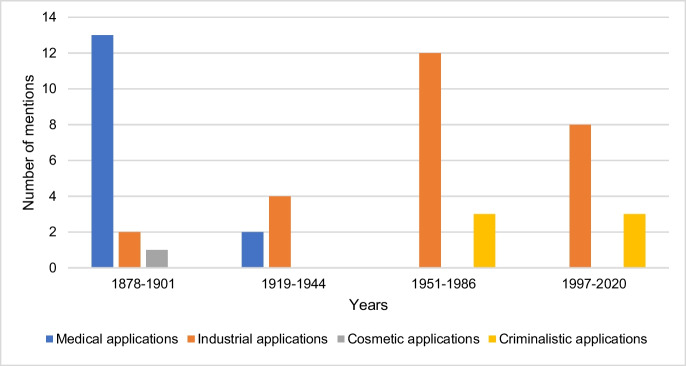
Information on the areas of application of hydrogen cyanide. The absolute number of mentions is shown

Figure 10 shows the information on the areas of application of hydrogen cyanide by textbook groups. The textbooks in the first textbook group list a total of three different areas of application for hydrogen cyanide, as medical, industrial, and cosmetic areas. The textbooks investigated in the second to fourth textbook group mention hydrogen cyanide applications in two different areas. The second textbook group contains information on medical and industrial applications, while the textbooks in the third and fourth textbook groups each list industrial and criminalistic uses of hydrogen cyanide. However, the use of hydrogen cyanide in the form of Zyklon B as a means of mass murder under National Socialism was only described in one textbook (16). Thus, most textbooks avoid dealing with the darkest episode of German history (and pharmacology), thereby missing an important opportunity to educate medical students and young physicians properly about ethics of pharmacology and toxicology.

### Acute symptoms of intoxication with hydrogen cyanide

**Fig. 11:**
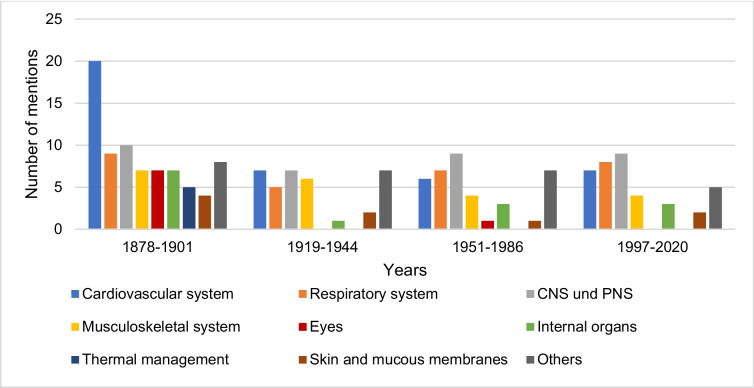
Information on the acute symptoms of hydrogen cyanide poisoning. The absolute number of mentions is shown

Figure 11 shows the data on the acute symptoms of hydrogen cyanide intoxication by textbook groups. The first textbook group list intoxication symptoms from nine systems. The information in the second and fourth textbook groups can each be assigned to seven systems. The acute intoxication symptoms listed in the third textbook group come from eight different systems. In the first textbook group, acute intoxication symptoms related to the cardiovascular system are mentioned most frequently. In the second textbook group, the *Cardiovascular system*, *CNS and PNS*, and *Other* account for most mentions. The textbooks in the third and fourth textbook groups most frequently mention symptoms of CNS and PNS poisoning.

### Lethal dose of hydrogen cyanide

**Fig. 12:**
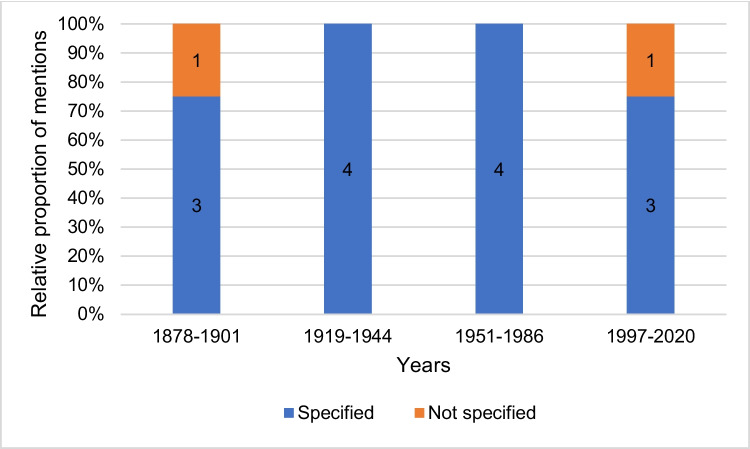
Data on the lethal dose of hydrogen cyanide. The number of entries per encoding is given as a percentage of the total number of entries per textbook group

Figure 12 shows how many textbooks provide information on the lethal dose of hydrogen cyanide. Among the textbooks investigated, just two do not contain any information on the lethal dose of hydrogen cyanide (2, 15).

**Fig. 13:**
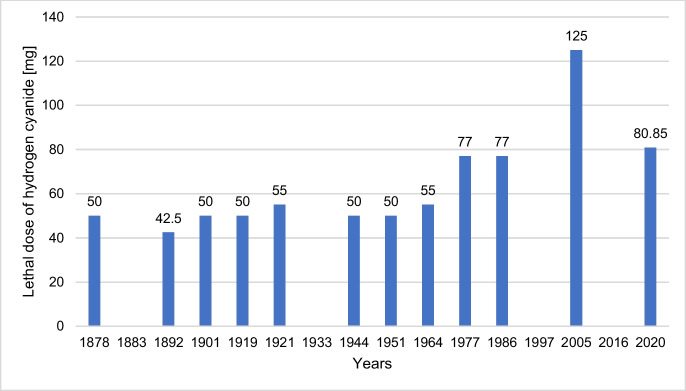
Lethal dose of HCN and CN^−^ in mg. Only listed lethal doses for adults are included in the evaluation. If there are two different listed doses per textbook, only the higher dose is shown in the figure. If the specified dosage has a range, the mean value is calculated and shown in the figure. Lethal doses in the unit mg/kg body weight are offset against the average German adult weight in 2021 (77 kg) and indicated (Statistisches Bundesamt, 2023, https://www.destatis.de/DE/Themen/Gesellschaft-Umwelt/Gesundheit/Gesundheitszustand-Relevantes-Verhalten/Tabellen/liste-koerpermasse.html)

Figure 13 shows the lethal doses for HCN and CN^−^ in mg. In the period 1878–1964, the lethal dose for hydrogen cyanide ranges between 42.5 and 55 mg. The textbooks for the period 1977–2020 describe lethal doses between 77 and 80 mg, 125 mg being an exception. In an analysis of the textbooks Lüllmann et al. (2003), Lemmer et al. (2004), and Scholz et al. (2005), the lethal doses given in 67% of the textbooks corresponded to the doses of the period 1878–1964. A similarly increased dose as in textbook 14 could only be found in Scholz et al. (2005). This supports the increased lethal dose in textbook 14 as an exception.

Thus, only a slight increase in the lethal doses from 1977 onwards can be observed, and the knowledge regarding the lethal dosage of hydrogen cyanide has remained almost constant.

### Treatment of hydrogen cyanide poisoning

**Fig. 14:**
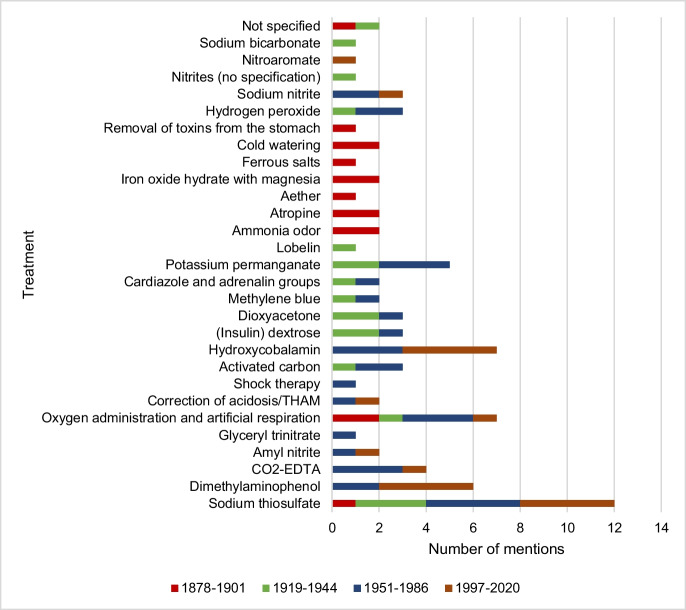
Information on the treatment of hydrogen cyanide poisoning. The absolute number of mentions is shown

Figure 14 shows the data on hydrogen cyanide treatment by textbook group. In the first textbook group, 50% of the textbooks describe oxygen administration and artificial respiration, ammonia odor, atropine, iron oxide hydrate with magnesia, and cold dousing as effective treatments for hydrogen cyanide poisoning. In the second and third textbook group, 75% and 100% of the textbooks provide information on sodium thiosulphate, which makes it the most mentioned treatment option in these textbook groups. The textbooks of the fourth textbook group categorize sodium thiosulfate, dimethylaminophenol, and hydroxycobalamine the most as treatment options.

From the second textbook group onwards, the focus of the textbooks is on treatment with sodium thiosulfate. With the start of the fourth textbook group in 1997, dimethylaminophenol and hydroxycobalamine also count as the most recommended treatment options.

### Recommended therapeutic preparations of hydrogen cyanide

**Fig. 15:**
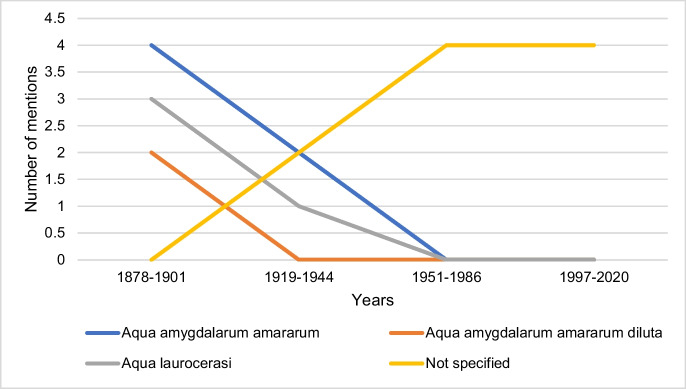
Information on recommended hydrogen cyanide preparations. The absolute number of entries is shown

Figure 15 shows the information on therapeutic preparations containing hydrogen cyanide. Most mentions come from the first textbook group, followed by a decline in the second textbook group. Textbooks in the third and fourth textbook groups did not provide any information in this category (see supplemental figure S10). This shows the decreasing therapeutic relevance of hydrogen cyanide.

Table 2 shows the single doses and daily doses given for the recommended therapeutic preparations in textbooks from 1878 to 1921. Most of the information is given for Aqua amygdalarum amararum, as 100% of the textbooks that recommend the preparation give dose details. A decrease in the maximum daily dose can be observed from 1901 onwards. For Aqua laurocerasi, only 75% of the textbooks that recommend the preparation provide information on dosage. Except for 1901, the doses mentioned are lower than for Aqua amygdalarum amararum. The dosage of Aqua amygdalarum amararum diluta is not mentioned in any textbook. Thus, the recommended daily doses of hydrogen cyanide remain 5–tenfold below lethal doses (Fig. 13), which is only a small safety margin. Probably, many accidental hydrogen cyanide intoxications occurred which was the reason for hydrogen cyanide being abandoned as a drug.

**Table 2:** Single and daily therapeutic doses of recommended hydrogen cyanide preparations. All textbooks that list hydrogen cyanide preparations are shown with the single and daily doses listed. Only the doses mentioned in the textbook itself are listed. Doses quoted from other textbooks are not shown. If a textbook has not specified single or daily doses, this is indicated in the table with n.m. for “not mentioned”

Years	**Aqua amygdalarum amararum**		**Aqua laurocerasi**		**Aqua amygdalarum amararum diluta**	
	Single dose	Daily dose	Single dose	Daily dose	Single dose	Daily dose
1878	n.m	6–20 drops	n.m	n.m	n.m	n.m
1883	0.5–2.0 mg	8.0 mg	n.m	n.m	n.m	n.m
1892	0.5–2.0 mg	8.0 mg	1.5 mg	5.0 mg	n.m	n.m
1901	2.0 mg	6.0 mg	2.0 mg	8.0 mg	n.m	n.m
1919	2.0 mg	6.0 mg	1.5 mg	5.0 mg	n.m	n.m
1921	0.5–2.0 mg	6.0 mg	n.m	n.m	n.m	n.m

## Discussion of incorrect content on hydrogen cyanide

**Fig. 16:**
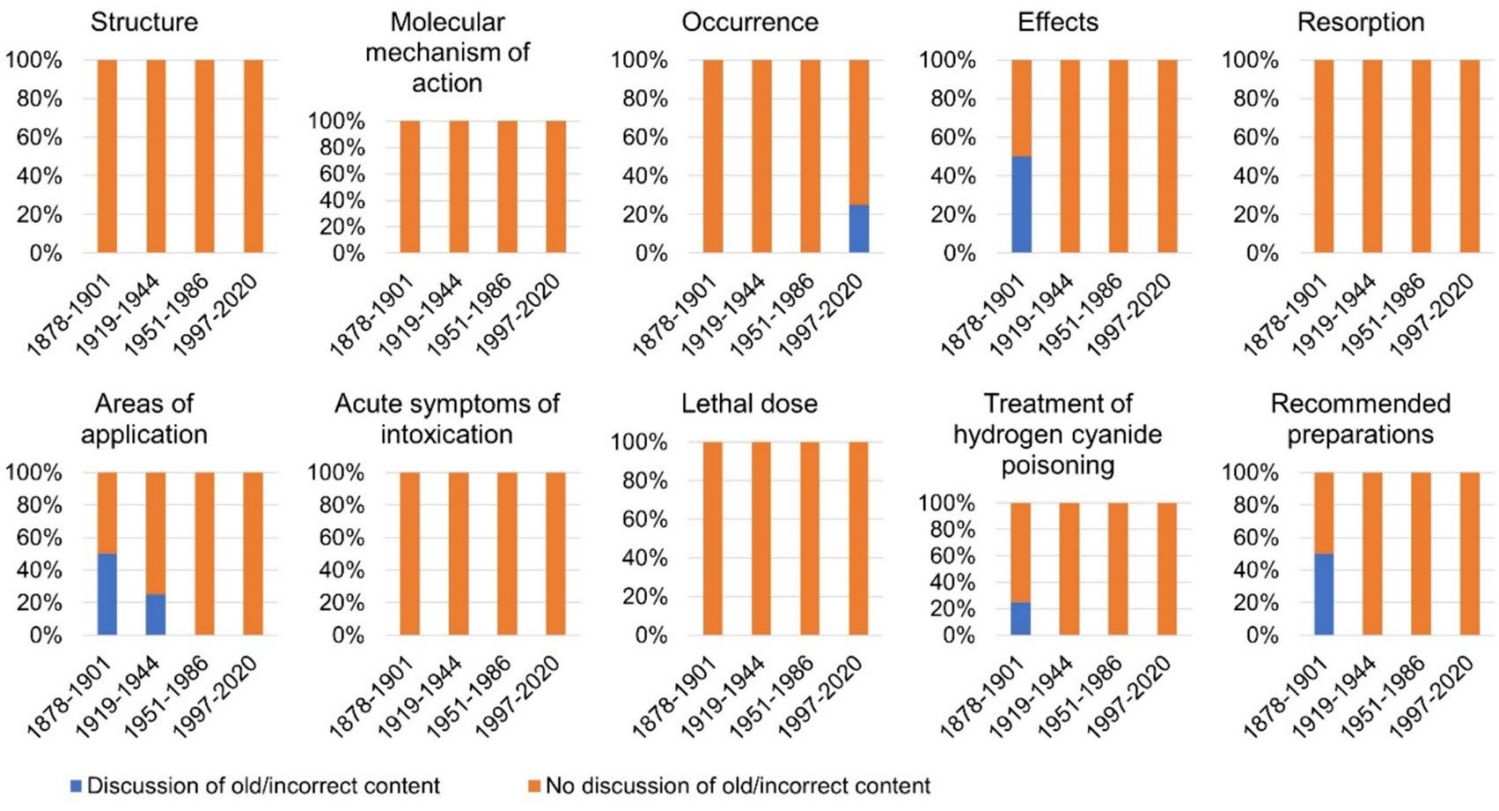
Discussion of incorrect content in pharmacological and toxicological textbooks. It was analyzed whether outdated and incorrect content is named and discussed as such in the textbooks. Only content that could be assigned to one of the ten analysis categories was included in the analysis. The proportion of textbooks in which incorrect content is discussed is given as a percentage of the total number of textbooks per textbook group

Figure 16 shows whether outdated and incorrect content is addressed as such in the pharmacology and toxicology textbooks. In the categories *Structure*, *Molecular mechanism of action*, *Resorption*, *Acute symptoms of intoxication*, and *Lethal dose*, 100% of the textbooks from the period 1878–2020 do not discuss any old or incorrect content. Incorrect contents of the categories *Effects* and *Recommended preparations* are discussed by 50% of the textbooks of the first textbook group. Twenty-five percent of the textbooks in the first textbook group discuss old content in the category *Treatment*. Incorrect content in the category *Areas of application* is discussed the most, as 50% of the textbooks in the first textbook group and 25% of the textbooks in the second textbook group address outdated knowledge. The category *Occurrence* is the only category in which outdated knowledge from modern textbooks from 1997 onwards is discussed, as 25% of the textbooks in the fourth textbook group address incorrect content on the occurrence of hydrogen cyanide. Thus, older textbooks are better at discussing advances in scientific concepts than newer textbooks. Newer textbooks tend to simply state current knowledge without discussing the dynamics of knowledge development. Thereby, modern textbooks mostly miss a great opportunity to educate medical students about the history of pharmacology and toxicology which is a history of change of concepts and facts as well as human trial and error.

## Limitations

Due to the restriction of the analysis to German-language pharmacology and toxicology textbooks, the transfer of the results to the international textbook literature is not guaranteed. Furthermore, we analyzed just one textbook per decade. The information on hydrogen cyanide in the textbooks by Schmiedeberg 1883 and 1921 (2 and 6), as well as by Eichholtz 1944 and 1951, (8 and 9) is similar. Since the key word index was used as the basis for the analysis and selection of the analyzed pages, only the information on hydrogen cyanide and its cyanides listed on the pages of the key word index was included in the analysis.

## Conclusions

Pharmacology and toxicology textbooks are used by students and doctors as a learning and reference tool, which is why they are of great importance for medical practice. Using hydrogen cyanide as a case study, we showed that over a period of 150 years, knowledge on chemical structure, lethal dose, and occurrence of hydrogen cyanide did not change much. In contrast, knowledge of the molecular mechanism of action, recommended preparations, effects, resorption, areas of application, acute symptoms of intoxication, and the treatment of hydrogen cyanide poisoning changed dramatically. From 1878 to 1901, primarily medical applications of the poison are described in the textbooks. From 1919, most of the areas of application listed are of industrial nature, and from 1951, there is an additional focus on criminal uses of the poison (murder, suicide, mass murder). The clinical obsolescence of the poison is reflected in the lack of mentions of medical applications in all recent textbooks. Accordingly, current textbooks (15, 16) are up to date regarding the areas of application. In contrast, with respect to reserpine, textbooks are lagging behind clinical practice (Misera and Seifert 2023). Thus, the validity of pharmacology and toxicology textbooks as a source for current information depends on the topic and drug.

The highest coverage of hydrogen cyanide in textbooks was found between 1933 and 1951 (7–9). Cyanide was used as “Zyklon B” in the Nazi concentration camps during the Second World War (1939–1945). However, in the pharmacology and toxicology textbooks published during the NS regime, no mentioning of the use of cyanide for mass murdering was made. Evidently, this criminal use of cyanide was kept secret, even in the textbook by Heinrich Gebhardt who was an active NSDAP and SS member (Philippu and Seifert 2023).

Based on the present study and the study by Misera and Seifert (2023), it will be worthwhile to analyze the presentation of other drugs and poisons in pharmacology and toxicology textbooks. Pharmacological and toxicological knowledge develops non-linearly for different aspects of a given drug, and it cannot be taken for granted that a current pharmacology and toxicology textbook provides current information. The case study of cyanide shows that pharmacology and toxicology have a long history of errors with respect to mechanism of action and clinical uses that were ultimately corrected but not discussed into a broader historical, ethical, societal or scientific context.

### What can be learned from the case study on hydrogen cyanide for the future of pharmacology and toxicology

It is often complained these days that the relevance of textbooks in general and textbooks of pharmacology and toxicology in particular is decreasing at the expense of powerpoint slides handed out in courses and commercially most successful platforms for answering multiple choice exam questions.

Our case study on hydrogen cyanide provides important strategies for how pharmacology and toxicology textbooks of the future can be made more appealing for students and, thereby, offering perspectives for textbook survival. New pharmacology and toxicology textbooks should take up the tradition of old textbooks, 100-150 years ago, and discuss how non-linearly and dynamically pharmacological  and toxicological knowledge evolves and what the reasons are behind such developments. Pharmacology and toxicology textbooks should also be more proactive at discussing the ethical and societal dimensions of pharmacology and toxicology. The case of hydrogen cyanide shows that this was an almost completely missed opportunity, an embarrassing neglect. These easily implemtable suggestions will sharpen the ability of critical thinking in medical students and provide them with important intellectual tools to shape the future of pharmacology and toxicology. Powerpoint slides and commercial collections of commented multiple choice questions cannot achieve this most intellectual important goal in student education. It is the responsibility of, and great opportunity for, textbook authors to reverse the increasing de-academization of pharmacological and toxicological education.

## Supplementary Information

Below is the link to the electronic supplementary material.

Supplementary file1 (DOCX 74 KB)

## Data Availability

All source data for this work are available upon reasonable request.

## Abbreviations

CN^−^: Cyanide
CNS: Central nervous system
Fe^3+^: Trivalent iron
HCN: Hydrogen cyanide
KCN: Potassium cyanide
kg: Kilogram
mg: Milligram
n.m.: Not mentioned
NaCN: Sodium cyanide
NS: National Socialism
NSDAP: National Socialist German Workers’ Party
PNS: Peripheral nervous system
SS: Schutzstaffel
Supp: Supplemented
