# Supplementary material for: How does pharmacological and toxicological knowledge evolve? A case study on hydrogen cyanide in German pharmacology and toxicology textbooks from 1878 to 2020
Source: Naunyn Schmiedebergs Arch Pharmacol. 2024 Jun 20;397(11):9167–80. doi: 10.1007/s00210-024-03227-z (PMC11522135; doi:10.1007/s00210-024-03227-z)
Supplement: Supplementary file 1 — Supplementary file1 (DOCX 74 KB) [file 210_2024_3227_MOESM1_ESM.docx]

**How does pharmacological knowledge evolve? A case study on hydrogen cyanide in German textbooks from 1878-2020**

**Laureen Ludwig and Roland Seifert**

**Supplemental figures**

| Coding | Structure | | 1878-1901 | 1919-1944 | 1951-1986 | 1997-2020 | All | Range |
| --- | --- | --- | --- | --- | --- | --- | --- | --- |
| 1 | Sum formula |  | 4 | 4 | 4 | 4 | 16 | 0 |
| 2 | Structural formula | | 0 | 0 | 0 | 0 | 0 | 0 |
| 3 | Not specified |  | 0 | 0 | 0 | 0 | 0 | 0 |
|  | Total mentions | | 4 | 4 | 4 | 4 | 16 |  |
|  | Average range | |  |  |  |  |  | 0 |

Figure S1: Category "Structure" with encodings. The frequency with which each encoding is represented per textbook group and the encoding´s range calculated from this is shown. In addition, the average range of encodings in this category was calculated.

| Coding | Molecular mechanism of action | | | | | | 1878-1901 | 1919-1944 | 1951-1986 | 1997-2020 | All | Range |
| --- | --- | --- | --- | --- | --- | --- | --- | --- | --- | --- | --- | --- |
| 1 | Binding to hemoglobin | |  |  |  |  | 3 | 2 | 1 | 0 | 6 | 3 |
| 2 | Binding to Fe3^+^ of the cytochrome oxidase | | | |  |  | 0 | 0 | 3 | 4 | 7 | 4 |
| 3 | Complex binding to the respiratory enzyme of the cell | | | |  |  | 0 | 1 | 0 | 0 | 1 | 1 |
| 4 | Not specified |  |  |  |  |  | 1 | 1 | 0 | 0 | 2 | 1 |
|  | Total mentions | |  |  |  |  | 3 | 3 | 4 | 4 | 14 |  |
|  | Average range | |  |  |  |  |  |  |  |  |  | 2.25 |

Figure S2: Category "Molecular mechanism of action" with encodings. The frequency with which each encoding is represented per textbook group and the encoding´s range calculated from this is shown. In addition, the average range of encodings in this category was calculated. The average range of the category is rounded to two decimal places.

| Coding | Occurrence | | | | | 1878-1901 | 1919-1944 | 1951-1986 | 1997-2020 | All | Range |
| --- | --- | --- | --- | --- | --- | --- | --- | --- | --- | --- | --- |
|  | Seeds |  |  |  |  | 7 | 6 | 10 | 8 | 31 |  |
| 1 | Bitter almonds | |  |  |  | 4 | 3 | 4 | 4 | 15 | 1 |
| 2 | Stone fruit kernels | |  |  |  | 3 | 3 | 3 | 2 | 11 | 1 |
| 3 | Pome seeds |  |  |  |  | 0 | 0 | 1 | 0 | 1 | 1 |
| 11 | Linseed |  |  |  |  | 0 | 0 | 2 | 2 | 4 | 2 |
|  | Plants and fungi | |  |  |  | 0 | 5 | 3 | 1 | 9 |  |
| 5 | Plants of the Lasia and Pangium species | | | |  | 0 | 1 | 0 | 0 | 1 | 1 |
| 6 | Juncaginaceen triglochin maritima and palustris | | | |  | 0 | 1 | 0 | 0 | 1 | 1 |
| 7 | Scheuchzeria palustris | |  |  |  | 0 | 1 | 0 | 0 | 1 | 1 |
| 8 | Indian moon beans | |  |  |  | 0 | 1 | 2 | 0 | 3 | 2 |
| 9 | Various mushrooms | |  |  |  | 0 | 1 | 0 | 0 | 1 | 1 |
| 12 | Manioc roots and fruits | |  |  |  | 0 | 0 | 1 | 1 | 2 | 1 |
|  | Smoke and combustion | |  |  |  | 0 | 2 | 7 | 5 | 13 |  |
| 13 | Tobacco smoke | |  |  |  | 0 | 1 | 3 | 1 | 5 | 3 |
| 14 | Incineration of hexamethylenetetramine | | |  |  | 0 | 0 | 2 | 0 | 2 | 2 |
| 15 | Incineration of polyacrylonitrile polymers, celluloid, wool or silk | | | | | 0 | 1 | 0 | 2 | 3 | 2 |
| 16 | Incineration of organic materials | | |  |  | 0 | 0 | 2 | 1 | 3 | 2 |
| 17 | Incineration of adhesive | |  |  |  | 0 | 0 | 0 | 1 | 1 | 1 |
|  | Pharmaceuticals | |  |  |  | 0 | 0 | 1 | 1 | 1 |  |
| 10 | Sodium nitroprusside | |  |  |  | 0 | 0 | 1 | 1 | 2 | 1 |
|  | Others |  |  |  |  | 1 | 0 | 2 | 2 | 5 |  |
| 4 | Nitriles |  |  |  |  | 1 | 0 | 2 | 2 | 5 | 2 |
| 18 | Not specified |  |  |  |  | 0 | 1 | 0 | 0 | 1 | 1 |
|  | Total mentions | |  |  |  | 8 | 13 | 23 | 17 | 59 |  |
|  | Average range | |  |  |  |  |  |  |  |  | 1.44 |

Figure S3: Category "Occurrence" with encodings. The frequency with which each encoding is represented per textbook group and the encoding´s range calculated from this is shown. In addition, the average range of encodings in this category was calculated. The average range of the category is rounded to two decimal places.

| Coding | Effects | | | | 1878-1901 | 1919-1944 | 1951-1986 | 1997-2020 | All | Range |
| --- | --- | --- | --- | --- | --- | --- | --- | --- | --- | --- |
| 1 | Excitation of the respiratory center | |  |  | 3 | 2 | 1 | 1 | 7 | 2 |
| 2 | Excitation of the vasomotor center and the vascular nerves | | | | 3 | 1 | 0 | 0 | 4 | 3 |
| 3 | Excitation of the convulsive center | |  |  | 2 | 1 | 0 | 0 | 3 | 2 |
| 4 | Excitation of the cardiac inhibitory fibers of the vagus nerve | | | | 2 | 0 | 0 | 0 | 2 | 2 |
| 5 | Inhibition of the respiratory center | |  |  | 2 | 1 | 1 | 0 | 4 | 2 |
| 6 | Inhibition of the vasomotor center and the vascular nerves | | | | 2 | 1 | 0 | 0 | 3 | 2 |
| 7 | Inhibition of the convulsive center | |  |  | 1 | 1 | 0 | 0 | 2 | 1 |
| 8 | Inhibition of the cardiac inhibitory fibers of the vagus nerve | | | | 1 | 0 | 0 | 0 | 1 | 1 |
| 9 | Inhibition of the motor cardiac ganglia | |  |  | 1 | 1 | 0 | 0 | 2 | 1 |
| 10 | Inhibited oxygen release from hemoglobin | | |  | 1 | 2 | 1 | 0 | 4 | 2 |
| 11 | Inhibited oxygen uptake and utilization in tissues | | |  | 2 | 2 | 4 | 4 | 12 | 2 |
| 12 | Inhibition of the respiratory chain and cellular oxidation processes | | | | 1 | 3 | 4 | 4 | 12 | 3 |
| 13 | Inhibition of ATP production | |  |  | 0 | 0 | 0 | 3 | 3 | 3 |
| 14 | (Cellular) oxygen deficiency/internal asphyxiation | | |  | 2 | 1 | 4 | 3 | 10 | 3 |
| 16 | Increase in saliva secretion | |  |  | 2 | 0 | 0 | 0 | 2 | 2 |
| 17 | Influence on metabolism (no specification) | | |  | 1 | 0 | 0 | 0 | 1 | 1 |
| 18 | Reduction in the excitability of peripheral muscles and nerves | | | | 1 | 0 | 0 | 0 | 1 | 1 |
| 19 | Activation of the chemoreceptors of the glomus caroticum | | | | 0 | 0 | 4 | 3 | 7 | 4 |
|  | Total mentions |  |  |  | 27 | 16 | 19 | 18 | 80 |  |
|  | Average range |  |  |  |  |  |  |  |  | 2.06 |

Figure S4: Category "Effects" with encodings. The frequency with which each encoding is represented per textbook group and the encoding´s range calculated from this is shown. In addition, the average range of encodings in this category was calculated. The average range of the category is rounded to two decimal places.

| Coding | Resorption | | | 1878-1901 | 1919-1944 | 1951-1986 | 1997-2020 | All | Range |
| --- | --- | --- | --- | --- | --- | --- | --- | --- | --- |
| 1 | Respiratory tract | |  | 2 | 1 | 4 | 3 | 10 | 3 |
| 2 | Gastrointestinal tract | |  | 3 | 2 | 4 | 4 | 13 | 2 |
| 3 | Skin |  |  | 2 | 1 | 1 | 1 | 5 | 1 |
| 4 | Eyes |  |  | 2 | 0 | 0 | 0 | 2 | 2 |
| 5 | Not specified |  |  | 1 | 2 | 0 | 0 | 3 | 2 |
|  | Total mentions | |  | 9 | 4 | 9 | 8 | 30 |  |
|  | Average range | |  |  |  |  |  |  | 2 |

Figure S5: Category "Resorption" with encodings. The frequency with which each encoding is represented per textbook group and the encoding´s range calculated from this is shown. In addition, the average range of encodings in this category was calculated.

| Coding | Areas of application | | | | 1878-1901 | 1919-1944 | 1951-1986 | 1997-2020 | All | Range |
| --- | --- | --- | --- | --- | --- | --- | --- | --- | --- | --- |
|  | Medical applications | |  |  | 13 | 2 | 0 | 0 | 15 |  |
| 1 | Flavor additive and enhancer | | |  | 3 | 2 | 0 | 0 | 5 | 3 |
| 2 | Gastralgia |  |  |  | 2 | 0 | 0 | 0 | 2 | 2 |
| 3 | Acid reflux |  |  |  | 1 | 0 | 0 | 0 | 1 | 1 |
| 4 | Analgesics |  |  |  | 2 | 0 | 0 | 0 | 2 | 2 |
| 5 | Asthma and breathing disorders | | |  | 2 | 0 | 0 | 0 | 2 | 2 |
| 6 | Beginning cardiac hypertrophy | | |  | 1 | 0 | 0 | 0 | 1 | 1 |
| 14 | Whooping cough in children <1 year | | |  | 1 | 0 | 0 | 0 | 1 | 1 |
| 15 | Pruritus |  |  |  | 1 | 0 | 0 | 0 | 1 | 1 |
|  | Industrial applications | |  |  | 2 | 4 | 12 | 8 | 26 |  |
| 7 | Pest control |  |  |  | 0 | 2 | 4 | 3 | 9 | 4 |
| 8 | Antiseptic agent | |  |  | 1 | 1 | 2 | 0 | 4 | 2 |
| 10 | Laboratories |  |  |  | 0 | 0 | 1 | 1 | 2 | 1 |
| 11 | Photography |  |  |  | 1 | 0 | 0 | 0 | 1 | 1 |
| 13 | Metal industry | |  |  | 0 | 1 | 4 | 4 | 9 | 4 |
| 16 | Silver polish |  |  |  | 0 | 0 | 1 | 0 | 1 | 1 |
|  | Cosmetic applications | |  |  | 1 | 0 | 0 | 0 | 1 |  |
| 12 | Perfume |  |  |  | 1 | 0 | 0 | 0 | 1 | 1 |
|  | Criminalistic applications | |  |  | 0 | 0 | 3 | 3 | 6 |  |
| 9 | Forensic use (mass murder, murder or suicide) | | | | 0 | 0 | 3 | 3 | 6 | 3 |
|  | Total mentions | |  |  | 16 | 6 | 15 | 11 | 48 |  |
|  | Average range | |  |  |  |  |  |  |  | 1.88 |

Figure S6: Category "Areas of application" with encodings. The frequency with which each encoding is represented per textbook group and the encoding´s range calculated from this is shown. In addition, the average range of encodings in this category was calculated. The average range of the category is rounded to two decimal places.

| Coding | Acute symptoms of intoxication | | | 1878-1901 | 1919-1944 | 1951-1986 | 1997-2020 | All | Range |
| --- | --- | --- | --- | --- | --- | --- | --- | --- | --- |
|  | Cardiovascular system | |  | 20 | 7 | 6 | 7 | 40 |  |
| 3 | Angina pectoris | |  | 3 | 1 | 0 | 0 | 4 | 3 |
| 4 | Palpitation |  |  | 2 | 0 | 1 | 0 | 3 | 2 |
| 5 | Negative chronotropy | |  | 2 | 0 | 0 | 0 | 2 | 2 |
| 6 | Positive chronotropy | |  | 2 | 0 | 0 | 0 | 2 | 2 |
| 7 | Heart failure and cardiac arrest | | | 3 | 2 | 0 | 0 | 5 | 3 |
| 8 | Increase in blood pressure | |  | 2 | 0 | 0 | 0 | 2 | 2 |
| 9 | Drop of blood pressure | |  | 2 | 0 | 0 | 0 | 2 | 2 |
| 10 | Arterialization of venous blood | | | 3 | 4 | 3 | 3 | 13 | 1 |
| 11 | Skin redness | |  | 0 | 0 | 2 | 3 | 5 | 3 |
| 36 | Metabolic acidosis | |  | 0 | 0 | 0 | 1 | 1 | 1 |
| 39 | Hemolysis |  |  | 1 | 0 | 0 | 0 | 1 | 1 |
|  | Respiratory system | |  | 9 | 5 | 7 | 8 | 29 |  |
| 14 | Breathing difficulties and respiratory paralysis | | | 4 | 3 | 4 | 3 | 14 | 1 |
| 15 | Increased respiratory rate | |  | 1 | 1 | 0 | 0 | 2 | 1 |
| 16 | Decreased respiratory rate | |  | 2 | 0 | 0 | 0 | 2 | 2 |
| 17 | Hyperpnea |  |  | 0 | 1 | 3 | 4 | 8 | 4 |
| 18 | Inspirational scream | |  | 2 | 0 | 0 | 1 | 3 | 2 |
|  | CNS und PNS | |  | 10 | 7 | 9 | 9 | 35 |  |
| 19 | Anxiety, confusion and impaired consciousness | | | 3 | 2 | 3 | 2 | 10 | 1 |
| 21 | Headaches and dizziness | |  | 3 | 2 | 2 | 3 | 10 | 1 |
| 22 | Unconsciousness and cardiovascular collapse | | | 2 | 3 | 3 | 3 | 11 | 1 |
| 23 | Coma |  |  | 2 | 0 | 0 | 1 | 3 | 2 |
| 40 | Intracerebral hemorrhage | |  | 0 | 0 | 1 | 0 | 1 | 1 |
|  | Musculoskeletal system | |  | 7 | 6 | 4 | 4 | 21 |  |
| 20 | Central or peripheral muscle paralysis and weakness | | | 2 | 3 | 1 | 0 | 6 | 3 |
| 27 | (Choking) convulsions and twitching | | | 4 | 2 | 3 | 3 | 12 | 2 |
| 28 | Trismus |  |  | 1 | 0 | 0 | 0 | 1 | 1 |
| 41 | Contraction/spasm of the muscles | | | 0 | 1 | 0 | 1 | 2 | 1 |
|  | Eyes |  |  | 7 | 0 | 1 | 0 | 8 |  |
| 29 | Mydriasis |  |  | 2 | 0 | 1 | 0 | 3 | 2 |
| 30 | Conjunctiva or iris changes | |  | 2 | 0 | 0 | 0 | 2 | 2 |
| 31 | Exophthalmos | |  | 2 | 0 | 0 | 0 | 2 | 2 |
| 42 | Burning eyes | |  | 1 | 0 | 0 | 0 | 1 | 1 |
|  | Internal organs | |  | 7 | 1 | 3 | 3 | 14 |  |
| 26 | Nausea and vomiting | |  | 2 | 1 | 3 | 2 | 8 | 2 |
| 33 | Urinary incontinence | |  | 2 | 0 | 0 | 0 | 2 | 2 |
| 34 | Fecal incontinence | |  | 2 | 0 | 0 | 0 | 2 | 2 |
| 38 | Feeling of warmth in the stomach | | | 1 | 0 | 0 | 0 | 1 | 1 |
| 44 | Red coloring of the urine | |  | 0 | 0 | 0 | 1 | 1 | 1 |
|  | Thermal management | |  | 5 | 0 | 0 | 0 | 5 |  |
| 12 | Increased body temperature | | | 2 | 0 | 0 | 0 | 2 | 2 |
| 13 | Decreased body temperature | | | 2 | 0 | 0 | 0 | 2 | 2 |
| 32 | Cold sweat |  |  | 1 | 0 | 0 | 0 | 1 | 1 |
|  | Skin and mucous membranes | | | 4 | 2 | 1 | 2 | 9 |  |
| 1 | Local anesthesia | |  | 3 | 2 | 0 | 0 | 5 | 3 |
| 2 | Local irritation of skin and mucous membranes (eye, nose, throat, dermis) | | | 1 | 0 | 1 | 2 | 4 | 2 |
|  | Others |  |  | 8 | 7 | 7 | 5 | 27 |  |
| 25 | Sore throat |  |  | 3 | 2 | 2 | 0 | 7 | 3 |
| 35 | Smell or taste of bitter almonds | | | 2 | 1 | 1 | 1 | 5 | 1 |
| 43 | Death |  |  | 3 | 4 | 4 | 4 | 15 | 1 |
|  | Total mentions | |  | 77 | 35 | 38 | 38 | 188 |  |
|  | Average range | |  |  |  |  |  |  | 1.79 |

Figure S7: Category "Acute symptoms of intoxication" with encodings. The frequency with which each encoding is represented per textbook group and the encoding´s range calculated from this is shown. In addition, the average range of encodings in this category was calculated. For better presentation, coding categories that could be assigned to two or more systems are only counted under one system. The average range of the category is rounded to two decimal places.

| Coding | Lethal dose | | 1878-1901 | 1919-1944 | 1951-1986 | 1997-2020 | All | Range |
| --- | --- | --- | --- | --- | --- | --- | --- | --- |
| 1 | Specified |  | 3 | 4 | 4 | 3 | 14 | 1 |
| 2 | Not specified |  | 1 | 0 | 0 | 1 | 2 | 1 |
|  | Total mentions | | 4 | 4 | 4 | 4 | 16 |  |
|  | Average range | |  |  |  |  |  | 1 |

Figure S8: Category "Lethal dose" with encodings. The frequency with which each encoding is represented per textbook group and the encoding´s range calculated from this is shown. In addition, the average range of encodings in this category was calculated.

| Coding | Treatment | | | | | 1878-1901 | 1919-1944 | 1951-1986 | 1997-2020 | All | Range |
| --- | --- | --- | --- | --- | --- | --- | --- | --- | --- | --- | --- |
| 1 | Sodium thiosulfate | |  |  |  | 1 | 3 | 4 | 4 | 12 | 3 |
| 2 | Dimethylaminophenol | |  |  |  | 0 | 0 | 2 | 4 | 6 | 4 |
| 3 | C_O2_-EDTA |  |  |  |  | 0 | 0 | 3 | 1 | 4 | 3 |
| 4 | Amyl nitrite |  |  |  |  | 0 | 0 | 1 | 1 | 2 | 1 |
| 5 | Glyceryl trinitrate | |  |  |  | 0 | 0 | 1 | 0 | 1 | 1 |
| 6 | Oxygen administration and artificial respiration | | | |  | 2 | 1 | 3 | 1 | 7 | 2 |
| 7 | Correction of acidosis/THAM | | |  |  | 0 | 0 | 1 | 1 | 2 | 1 |
| 8 | Shock therapy | |  |  |  | 0 | 0 | 1 | 0 | 1 | 1 |
| 9 | Activated carbon | |  |  |  | 0 | 1 | 2 | 0 | 3 | 2 |
| 10 | Hydroxycobalamin | |  |  |  | 0 | 0 | 3 | 4 | 7 | 4 |
| 11 | (Insulin) dextrose | |  |  |  | 0 | 2 | 1 | 0 | 3 | 2 |
| 12 | Dioxyacetone | |  |  |  | 0 | 2 | 1 | 0 | 3 | 2 |
| 13 | Methylene blue | |  |  |  | 0 | 1 | 1 | 0 | 2 | 1 |
| 14 | Cardiazole and adrenalin groups | | |  |  | 0 | 1 | 1 | 0 | 2 | 1 |
| 15 | Potassium permanganate | |  |  |  | 0 | 2 | 3 | 0 | 5 | 3 |
| 16 | Lobelin |  |  |  |  | 0 | 1 | 0 | 0 | 1 | 1 |
| 17 | Ammonia odor | |  |  |  | 2 | 0 | 0 | 0 | 2 | 2 |
| 18 | Atropine |  |  |  |  | 2 | 0 | 0 | 0 | 2 | 2 |
| 19 | Aether |  |  |  |  | 1 | 0 | 0 | 0 | 1 | 1 |
| 20 | Iron oxide hydrate with magnesia | | |  |  | 2 | 0 | 0 | 0 | 2 | 2 |
| 21 | Ferrous salts | |  |  |  | 1 | 0 | 0 | 0 | 1 | 1 |
| 22 | Cold watering | |  |  |  | 2 | 0 | 0 | 0 | 2 | 2 |
| 23 | Removal of toxins from the stomach | | |  |  | 1 | 0 | 0 | 0 | 1 | 1 |
| 24 | Hydrogen peroxide | |  |  |  | 0 | 1 | 2 | 0 | 3 | 2 |
| 25 | Sodium nitrite | |  |  |  | 0 | 0 | 2 | 1 | 3 | 2 |
| 26 | Nitrites (no specification) | |  |  |  | 0 | 1 | 0 | 0 | 1 | 1 |
| 27 | Nitroaromate |  |  |  |  | 0 | 0 | 0 | 1 | 1 | 1 |
| 28 | Sodium bicarbonate |  |  |  |  | 0 | 1 | 0 | 0 | 1 | 1 |
| 29 | Not specified |  |  |  |  | 1 | 1 | 0 | 0 | 2 | 1 |
|  | Total mentions | |  |  |  | 14 | 16 | 32 | 18 | 80 |  |
|  | Average range | |  |  |  |  |  |  |  |  | 1.76 |

Figure S9: Category "Treatment" with encodings. The frequency with which each encoding is represented per textbook group and the encoding´s range calculated from this is shown. In addition, the average range of encodings in this category was calculated. The average range of the category is rounded to two decimal places.

| Coding | Recommended preparations | | |  | 1878-1901 | 1919-1944 | 1951-1986 | 1997-2020 | All | Range |
| --- | --- | --- | --- | --- | --- | --- | --- | --- | --- | --- |
| 1 | Aqua amygdalarum amararum | | |  | 4 | 2 | 0 | 0 | 6 | 4 |
| 2 | Aqua amygdalarum amararum diluta | | |  | 2 | 0 | 0 | 0 | 2 | 2 |
| 3 | Aqua laurocerasi | |  |  | 3 | 1 | 0 | 0 | 4 | 3 |
| 4 | Not specified |  |  |  | 0 | 2 | 4 | 4 | 10 | 4 |
|  | Total mentions | |  |  | 9 | 3 | 0 | 0 | 12 |  |
|  | Average range | |  |  |  |  |  |  |  | 3.25 |

Figure S10: Category "Recommended preparations" with encodings. The frequency with which each encoding is represented per textbook group and the encoding´s range calculated from this is shown. In addition, the average range of encodings in this category was calculated. The average range of the category is rounded to two decimal places.
